# Objective measurement of gait parameters in healthy and cognitively impaired elderly using the dual-task paradigm

**DOI:** 10.1007/s40520-016-0703-6

**Published:** 2017-01-27

**Authors:** Alexandra König, Laura Klaming, Marten Pijl, Alexandre Demeurraux, Renaud David, Philippe Robert

**Affiliations:** 1 CoBTeK Research Unit, University of Nice, Sophia Antipolis, France; 2 Philips Research, Eindhoven, The Netherlands; 3 Centre Mémoire de Ressources et de Recherche, CHU de Nice, Nice, France

**Keywords:** Dementia, Alzheimer, Mild cognitive impairment, Motor function, Gait, Actigraphy, Accelerometer, Dual-task, Attention

## Abstract

**Objectives:**

The present study explores the differences in gait parameters in elderly subjects with or without cognitive impairment measured by means of ambulatory actigraphy while performing a single and a dual task.

**Methods:**

Sixty-nine participants of which 23 individuals were diagnosed with Alzheimer’s disease (AD), 24 individuals with mild cognitive impairment (MCI), and 22 healthy controls performed a single and dual walking task while wearing a wrist-worn accelerometer. Objective measures of gait features such as walking speed, cadence (i.e., number of steps per minute), and step variance (i.e., variance in time between two consecutive steps) were derived and analyzed.

**Results:**

While differences in several gait parameters, namely walking speed, were found between MCI and AD patients, no differences between healthy elderly and MCI patients were found.

**Conclusion:**

Walking speed seems to be a gait-related feature that differs significantly between MCI and AD patients and thus could be used as an additional measurement in clinical assessment. However, differences in gait may not be salient enough in the early stages of dementia to be detected by actigraphy. More research comparing different methods to measure gait in early stages of dementia under different dual task conditions is neccessary.

## Introduction

Alzheimer’s disease (AD) is the most common neurodegenerative disorder and one of the leading causes of death at old age [1, 2]. AD affects different domains of functioning, including cognitive and motor functioning [2, 3]. Motor functioning involves the integration of various cognitive functions including visuospatial perception, attention, and planning. Deficits in these cognitive functions can therefore affect motor functioning, and subtle changes in motor functioning could be an early indicator of cognitive decline [4]. Identifying patients in pre-dementia states, such as mild cognitive impairment (MCI), is an important clinical need since treatment may be more effective in early stages [1, 5]. This has led to an increase in research on early markers for cognitive decline, including motor markers [4]. The relation between motor activity and dementia has received increasing research attention over the past years [4, 6–24]. Studies have shown that compared to healthy elderly, AD patients walk more slowly and have an increased fall risk [9]. In addition, research has shown that gait disturbances can be observed in early AD and can predict progression from MCI to AD [7, 8, 11, 12, 20]. Research on gait disturbances as an early indicator for MCI and AD often uses the dual-task paradigm to explore the influence of cognitive functioning on motor functioning [4, 6, 15–19]. Dual tasking relies on dividing attention between two distinct tasks, often a motor task such as walking and a cognitively demanding task such as counting backwards. The dual-task paradigm can be used to study the allocation of attentional resources during a motor task and to separate the cognitive and motor components of executing a movement [4]. MCI and AD patients typically show more pronounced decrements in gait when performing two tasks simultaneously compared to healthy elderly [15, 16, 19]. Different cognitive tasks have been used for dual tasking including simple tasks such as counting backwards [6, 16], or more complex tasks such as a verbal fluency [6, 16] or reciting alternate letters of the alphabet [17]. Generally, increasing cognitive effort in the dual task increases sensitivity of the gait assessment but in older adults an attention-demanding arithmetic task such as counting backwards seems to be more appropriate for gait analyses [18].

Several studies have explored gait-related features such as speed of walking, stride frequency, and length and symmetry of steps in elderly with and without cognitive impairment during the performance of a single and a dual task [4, 6, 12, 15, 16, 18, 19]. In a study including 14 MCI patients, six AD patients, and 14 healthy control subjects, participants were asked to walk a distance of 45 m, while gait parameters were measured by means of two actigraphs attached at the participants’ waist [15]. AD patients were found to be slower than MCI patients who were found to be slower than healthy controls during the dual task involving walking and counting backwards. Additionally, MCI and mild AD patients showed deviations in other aspects of gait during the dual task compared to the single task, which were not found in healthy controls. Other studies that have only included MCI patients and not AD patients have found similar results [4, 6, 13, 16]. For instance, a study including 55 MCI patients found that participants walked significantly slower during a dual task than during a single task [6]. Similarly, a study measuring differences in in-home walking trajectories between 31 non-amnestic MCI patients and 54 healthy elderly found that individuals with non-amnestic MCI walked slower than healthy elderly [13]. Additionally, non-amnestic MCI patients showed a more pronounced decrease in walking speed variability over time than healthy controls whose walking speed variability remained stable. This finding suggests that walking speed and the variation in walking speed over time may be an early marker of MCI even at a state when memory functions are still intact [13]. Similarly, Beauchet et al. investigated stride velocity and stride-to-stride variability of stride time in 39 MCI patients, 33 AD patients, and 44 healthy elderly finding that both gait features increased during a dual-task condition compared to a single-task condition [12]. Moreover, stride-to-stride variability was found to be greater in MCI patients than in healthy elderly and AD patients in fast-pace walking suggesting that it is a specific feature of MCI under a fast-pace walking condition [12].

A possible explanation for the finding that individuals with cognitive decline show disturbances in their gait, particularly under dual-task conditions, is neuropathological changes in specific brain regions involved in the planning and execution of movements and that occur in early stages of dementia [2, 4, 21]. For instance, one study found a correlation between specific gait parameters (gait velocity, stride time variability) and the cerebral volume of the motor area as well as the presence of neurochemical changes in MCI patients during single and dual tasking [21].

Together, the findings of the studies described above suggest that changes in walking parameters, such as walking speed and variability in stride time, can be detected in early stages of cognitive decline and can therefore be a biomarker of MCI. Research has only recently started to look into ways to measure the link between cognitive and motor function and to more objectively detect subtle changes that could indicate MCI or progression from MCI to AD. Sensing technologies are used more and more to monitor and assess motor behavior in elderly people [25]. Most of the studies on gait in MCI and AD patients have employed pressure-point systems, such as GAITRite® System [4, 10–12, 17, 19, 21], or passive infrared sensors [13] which are not always affordable for all clinical sites. A more practical and low-cost solution for gait analysis is ambulatory actigraphy which consists of a piezoelectric accelerometer designed to record body movements. Actigraphy has previously been used in the assessment of various disorders including sleep–wake disorders, hyperactivity disorders, and dementia [22–24, 26–28]. The present study aims at exploring the relation between gait parameters, measured by means of ambulatory actigraphy during a single and dual task, and cognitive impairment in order to obtain more insights into the utility of such a paradigm as an additional indicator for the diagnosis of MCI and early AD.

## Materials and methods

This study was conducted within the European project Dem@care which aims at developing a multiple sensor-based system to assess specific behaviors of people with dementia and to provide feedback to patients, caregivers, and clinicians.

### Participants and procedure

Participants aged 65 years or older were recruited within the Dem@care protocol [29] at the Nice Memory Research Center located at the Geriatric Department of the University Hospital. The sample consisted of 24 individuals diagnosed with MCI, 23 individuals diagnosed with AD, and 22 healthy controls (HC). For the AD group, the diagnosis was determined using the proposed diagnostic criteria from Dubois et al. [30] requiring the presence of a progressive episodic memory impairment and biomarker evidence. For the MCI group, patients were diagnosed using the Petersen clinical criteria [31]. In addition, subjects were required to have a mini-mental state examination (MMSE) [32] score higher than 24. Subjects were not included if they had a history of head trauma with loss of consciousness, history of lower limb surgery, arthritis, obesity (BMI higher than 30), concomitant medication including benzodiazepines or antipsychotics, psychotic or aberrant motor activity (tremor, rigidity, Parkinsonism) as defined by the Movement Disorder Society Unified Parkinson Disease Rating Scale [33] in order to control for any possible motor disorders influencing the ability to carry out a walking task. The study was approved by the local ethics committee of the geriatric hospital in Nice, and only participants with the capacity to consent to the study were included. Each participant gave informed consent prior to the study. The consent was only given to publish demographic and accelerometer data but no biomarker data.

### Assessments and clinical protocol

All participants performed a single walking task (ST) that consisted of walking 10 m, turning around and walking 10 m backwards. Subsequently, all participants performed a dual task (DT) that involved walking the same distance while counting backwards from 305 in steps of 1. All participants performed the tasks in the same corridor in the Memory clinic, which is approximately 10 m long and 2 m wide. The corridor had normal daylight and a stable room temperature of 25 °C. During both tasks, participants wore a wrist-worn accelerometer from which objective measures for walking speed, cadence (i.e., number of steps per minute), and step variance (i.e., variance in time between two consecutive steps) were derived. The accelerometer data were analyzed by determining segments of walking data from the raw signal, and by applying step detection using a step detection algorithm that selects steps based on peaks in the accelerometer magnitude signal using a set of heuristics related to the time between consecutive steps and the amplitudes of the signal peaks. Neuropsychological measures included the MMSE [31], frontal assessment battery (FAB) [34], and trail making test (TMT) A and B [35].

### Motion data acquisition and analysis

Gait was measured using a CE-marked accelerometer research prototype (developed by Philips Research Laboratories Europe), a wrist-worn device containing a 3D accelerometer and data storage capabilities. The accelerometer was worn by the participants for the duration of the trial, after which the actigraphy data were retrieved from the device by the experimenter. During the trial, the experimenter indicated the start and end of both the ST and DT condition by pressing an event button on the accelerometer, creating an annotation on the device such that actigraphy data from both tasks could be easily extracted from the recording.

After extracting the actigraphy data, each recording was linked to the participant through a participant ID, and the actigraphy data for the individual ST and DT were extracted using the event markers recorded by the device. The actigraphy data for the tasks were then further cleaned by removing any initial and trailing periods of inactivity, caused by, e.g. the delay between the creation of the event marker and the commencement of the actual task.

Gait features were then determined algorithmically, using a heuristics-based step detection algorithm. The algorithm involves cleaning the accelerometer signal with a bandpass filter, finding a number of peaks in the filtered signal as potential steps, and creating a selection of the detected peaks which optimize a set of heuristic rules regarding the peak amplitude and distance to other peaks. From the detected steps, cadence was derived as the number of steps per minute, and step variance as the variance of the time between successive steps. Walking speed was derived as the distance traveled, divided by the time between the first and last step. Walking speed was derived as the distance traveled, divided by the time between the first and last step. A more detailed explanation of the algorithm and its performance on a previous dataset can be found in [36].

### Statistical analysis

Statistical analysis was performed using SPSS 23. Analyses included Chi-square test, one-way analysis of variance (ANOVA), mixed between-within subjects ANOVA, and correlation analyses. Post hoc tests were performed with Bonferroni correction.

## Results

### Demographics and clinical assessments

The study included a total of 69 participants of which 23 individuals were diagnosed with AD (mean age = 77 years ± 9, MMSE = 17 ± 4.6), 24 individuals were diagnosed with MCI (mean age = 75 ± 9, MMSE = 24.8 ± 3.2), and 22 were healthy controls (mean age = 73 ± 7, MMSE = 28.4 ± 1.5). Demographic information and neuropsychological test results for the three groups are presented in Table 1.

**Table 1:** Demographic information and neuropsychological tests for three groups

	Gender (male/female)	Age	MMSE	FAB	TMT A (in s)	TMT B (in s)
HC	5/15	73 (*SD* = 7)	28.35 (*SD* = 1.5)	15.94 (*SD* = 1.78)	45.38 (*SD* = 15.2)	118 (*SD* = 45.7)
MCI	8/16	75 (*SD* = 9)	24.75 (*SD* = 3.18)	15.1 (*SD* = 1.74)	56.4 (*SD* = 19.1)	171.73 (*SD* = 94.78)
AD	12/11	77 (*SD* = 9)	17 (*SD* = 4.62)	10.89 (*SD* = 3.94)	66.58 (*SD* = 37.67)	279.29 (*SD* = 64.05)

*HC* healthy control subjects, *MCI* mild cognitive impairment subjects, *AD* Alzheimer’s disease subjects, *MMSE* mini-mental state examination, *FAB* frontal assessment battery, *TMT A* trail making test version A, *TMT B* trail making test version B

There was no significant difference between the three groups in gender [χ²(2, 67) = 3.63, *p* = .163] or age [F(2, 66) = 1.63, *p* = .204]. Information about the MMSE was available for 67 participants. As expected, individuals diagnosed with AD had a lower MMSE [*N* = 23, mean = 17 (± 4.62)] than individuals diagnosed with MCI and HC, and individuals diagnosed with MCI [*N* = 24, mean = 24.75 (± 3.18)] had a lower MMSE than HC [*N* = 20, mean = 28.35 (± 1.5)]. All differences were statistically significant [F(2,66) = 63.23, *p* < .0001]. Information about the different subscales of the MMSE [24] was available for 47 participants.^1^ As shown in Table 2, the differences between the HC and MCI are rather small and the differences between the HC and AD seem to be particularly pronounced in the temporal, attention and calculation, and recall subscores. A one-way ANOVA revealed significant differences for all subscales^2^ (orientation in time: F(2,46) = 24.47, *p* < .0001; orientation in place: F(2,46) = 22.1, *p* < .0001; registration: F(2,46) = 4.17, *p* = .022; attention and calculation: F(2,46) = 11.56, *p* < .0001; recall: F(2,46) = 23.52, *p* < .0001; language: F(2,46) = 9.24, *p* < .0001; and complex commands: F(2,45) = 7.25, *p* = .002). Post hoc tests revealed a significant difference between HC and AD (*p* < .0001) and MCI and AD (*p* < .0001) for the orientation in time subtest, between the HC and AD (*p* < .0001) and MCI and AD (*p* < .0001) for the orientation in place subtest, between MCI and AD (*p* = .033) for the registration subtest, between HC and AD (*p* < .0001) and MCI and AD (*p* = .008) for the attention and calculation subtest, between HC and MCI (*p* = .001), between HC and AD (*p* < .0001) and between MCI and AD (*p* = .003) for the recall subtest, between HC and AD (*p* = .002) and MCI and AD (*p* = .002) for the language subtest, and between HC and AD (*p* = .013) and MCI and AD (*p* = .003) for the complex commands subtest  (Table 3).

**Table 2:** Scores on MMSE subscales for three groups

	Orientation in time	Orientation in place	Registration	Attention and calculation	Recall	Language	Complex commands
HC	5 (*SD* = 0)	5 (*SD* = 0)	3 (*SD* = 0)	4.55 (*SD* = 0.82)	2.91 (*SD* = 0.3)	7.64 (*SD* = 0.67)	1 (*SD* = 0)
MCI	4.25 (*SD* = 1.48)	4.25 (*SD* = 0.91)	3 (*SD* = 0)	3.35 (*SD* = 1.6)	1.6 (*SD* = 1)	7.45 (*SD* = 0.61)	1 (*SD* = 0)
AD	1.94 (*SD* = 1.29)	2.75 (*SD* = 1.18)	2.69 (*SD* = 0.6)	1.69 (*SD* = 1.85)	0.56 (*SD* = 0.96)	6.56 (*SD* = 0.89)	0.67 (*SD* = 0.49)

*HC* healthy control subjects, *MCI* mild cognitive impairment subjects, *AD* Alzheimer’s disease subjects, *MMSE* mini-mental state examination, *SD* standard deviation

**Table 3:** Walking speed, cadence and step variance for three groups

	Walking speed ST (in sec)	Walking speed DT (in sec)	Cadence ST (steps/min)	Cadence DT (steps/min)	Step variance ST	Step variance DT
HC	22.62 (*SD* = 3.03)	26.46 (*SD* = 6.4)	101.57 (*SD* = 12.69)	95.98 (*SD* = 14.03)	0.045 (*SD* = 0.049)	0.039 (*SD* = 0.054)
MCI	25.88 (*SD* = 7.7)	30.95 (*SD* = 10)	99.95 (*SD* = 8.99)	87.28 (*SD* = 14.18)	0.057 (*SD* = 0.045)	0.068 (*SD* = 0.053)
AD	26.34 (*SD* = 5.75)	31.91 (*SD* = 7.79)	97.19 (*SD* = 11.06)	84.84 (*SD* = 13.44)	0.067 (*SD* = 0.071)	0.102 (*SD* = 0.099)

*HC* healthy control subjects, *MCI* mild cognitive impairment subjects, *AD* Alzheimer’s disease subjects, *MMSE* mini-mental state examination, *ST* single-task condition, *DT* dual-task condition

Information about the FAB was available for 55 participants. Post hoc tests showed that participants diagnosed with AD [*N* = 18, mean = 10.89 (± 3.94)] had significantly lower scores on the FAB than individuals diagnosed with MCI [*N* = 20, mean = 15.1 (± 1.74), F(2,54) = 18.32, *p* < .0001] and HC [*N* = 17, mean = 15.94 (± 1.78), F(2,54) = 18.32, *p* < .0001].

Information about the TMT was available for 46 participants for version A and for 39 participants for version B. Information about the TMT A was available for 15 AD patients of whom three took so long that they were not asked to perform version B and who were therefore excluded from the analyses. When excluding these three patients, there was no difference between the three groups in time needed to perform version A of the TMT (F(2,42) = 2.58, *p* = .088). A one-way ANOVA did, however, find a difference between groups for the TMT B [F(2,37) = 12.22, *p* < .0001]. Post hoc tests revealed that AD patients [*N* = 7, mean = 279.29 s, (± 64.05 s)] needed significantly longer to complete the TMT B than both MCI patients [*N* = 15, mean = 171.73 s, (± 94.78 s), *p* = .007] and HC [*N* = 16, mean = 118 s, (± 45.7 s), *p* < .0001].

**Fig. 1:**
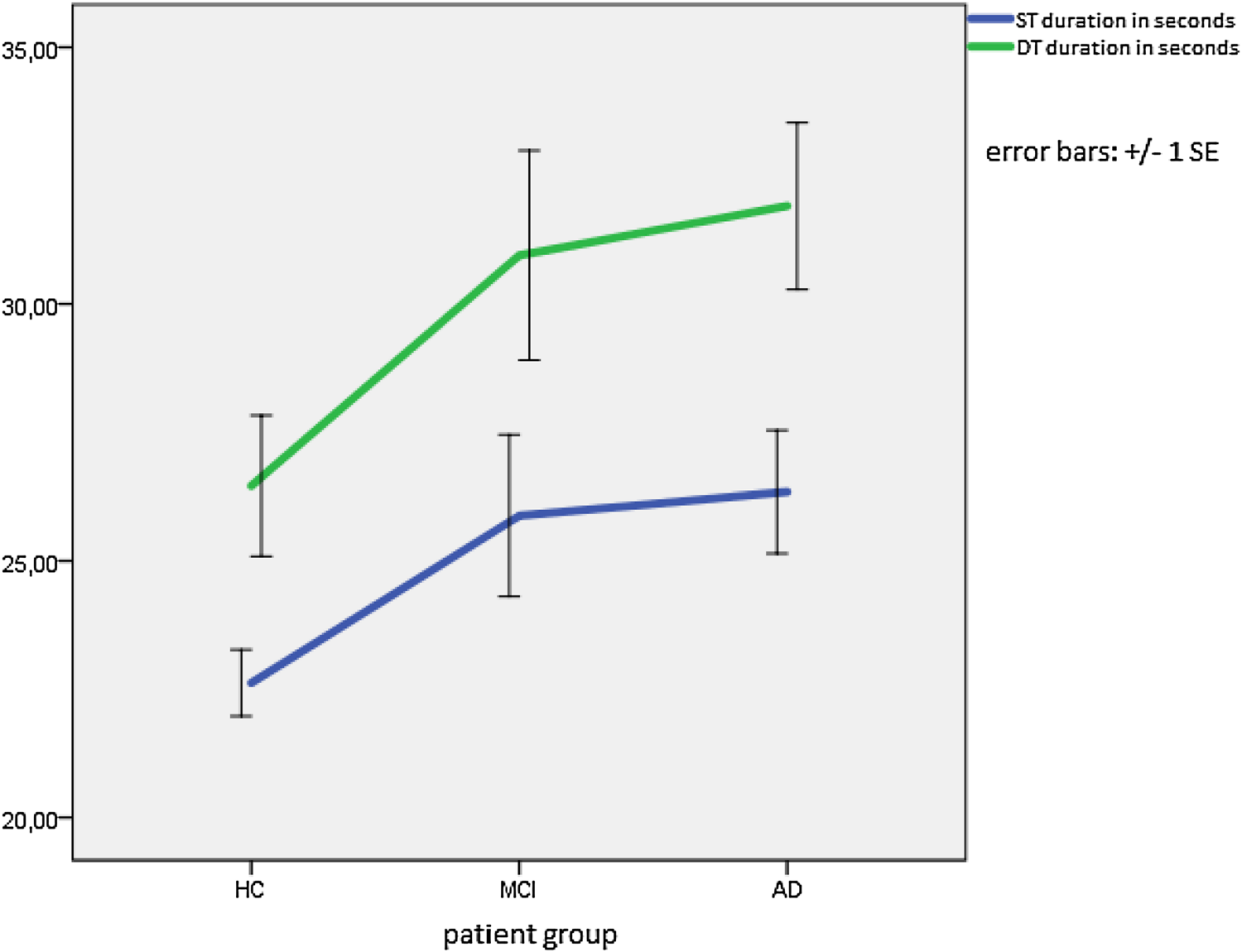
Duration (in seconds indicated on the Y axis) speed during the ST (*blue*) and the DT (*green*). *HC* healthy control subjects, *MCI* mild cognitive impairment subjects, *AD* Alzheimer’s disease subjects, *ST* single-task condition, *DT* dual-task condition

All participants were slower during the DT than during the ST (see Fig. 1). Interestingly, there seems to be a steeper increase in duration (i.e., a decrease in walking speed) from HC to MCI than from MCI to AD for both the ST and the DT. A mixed between-within ANOVA found a significant main effect for walking speed (Wilks’s Lambda = 0.76, F(1,66) = 20.89, *p* < .0001, partial eta squared = 0.24) with all groups showing a difference in walking speed between the ST and the DT. The difference between groups was significant [F(1,66) = 4.24, *p* = .019, partial eta squared = 0.114]. Post-hoc tests revealed that the difference in walking speed between the ST and DT differed between the HC [22.62 (± 3.03) vs. 26.46 (± 6.42)] and the AD group [26.34 (± 5.74) vs. 31.91 (± 7.79), *p* = .026] with the increase in duration (i.e., the decrease in walking speed) from the ST to the DT being greater for the AD patients. Although the increase in duration (i.e., the decrease in walking speed) from ST to DT was also greater for MCI [25.88 (± 7.7) vs. 30.95 (± 10)] patients than for HC, the difference between these two groups failed to reach significance (*p* = .072). No correlations were found between DT duration and neuropsychological measures of aspects of attention such as the MMSE subscale attention and calculation (*r*  =−.19) and the TMT B (*r*  = .294) or measures of motor performance such as the MMSE subscale complex commands (*r* =−.029).

**Fig. 2:**
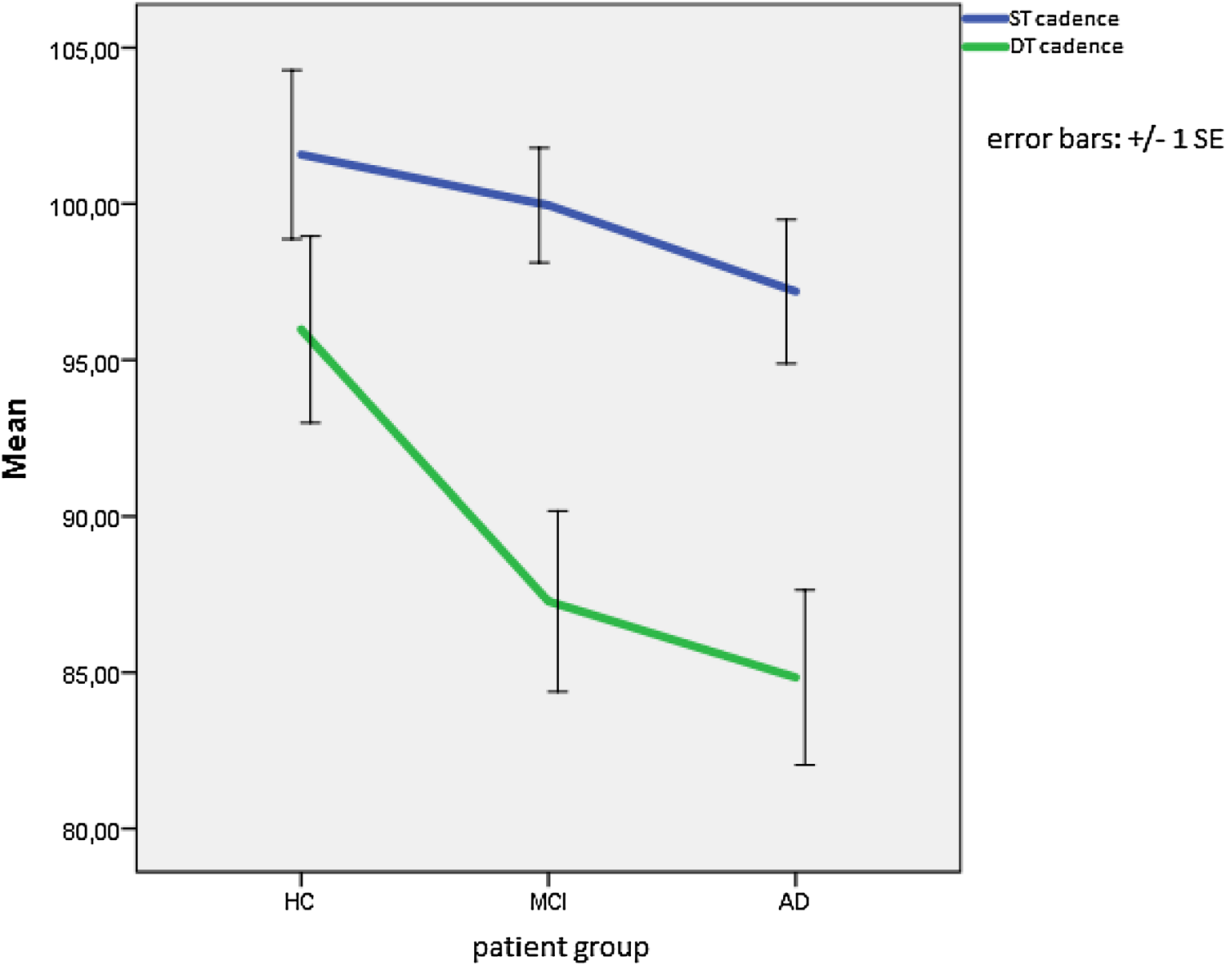
Cadence (number of steps per minute indicated on the *Y axis*) during the ST (*blue*) and the DT (*green*). *HC* healthy control subjects, *MCI* mild cognitive impairment subjects, *AD* Alzheimer’s disease subjects, *ST* single-task condition, *DT* dual-task condition

All participants had a lower cadence during the DT than the ST (see Fig. 2). The difference in cadence between the ST and the DT is more pronounced for the MCI and AD patients than for the HC. A mixed between-within ANOVA found a significant main effect for cadence [Wilks’s Lambda = 0.57, F(1,66) = 50.28, *p* < .0001, partial eta squared = 0.432] with all groups showing a difference in cadence between the ST and the DT. The difference between groups did not reach statistical significance [F(2,66) = 2.89, *p* = .062, partial eta squared = 0.081]. No or low correlations were found between DT cadence and neuropsychological measures of aspects of attention such as the MMSE subscale attention and calculation (*r* = .125) and the TMT B (*r* = −326) or measures of motor performance such as the MMSE subscale complex commands (*r* = .037).

**Fig. 3:**
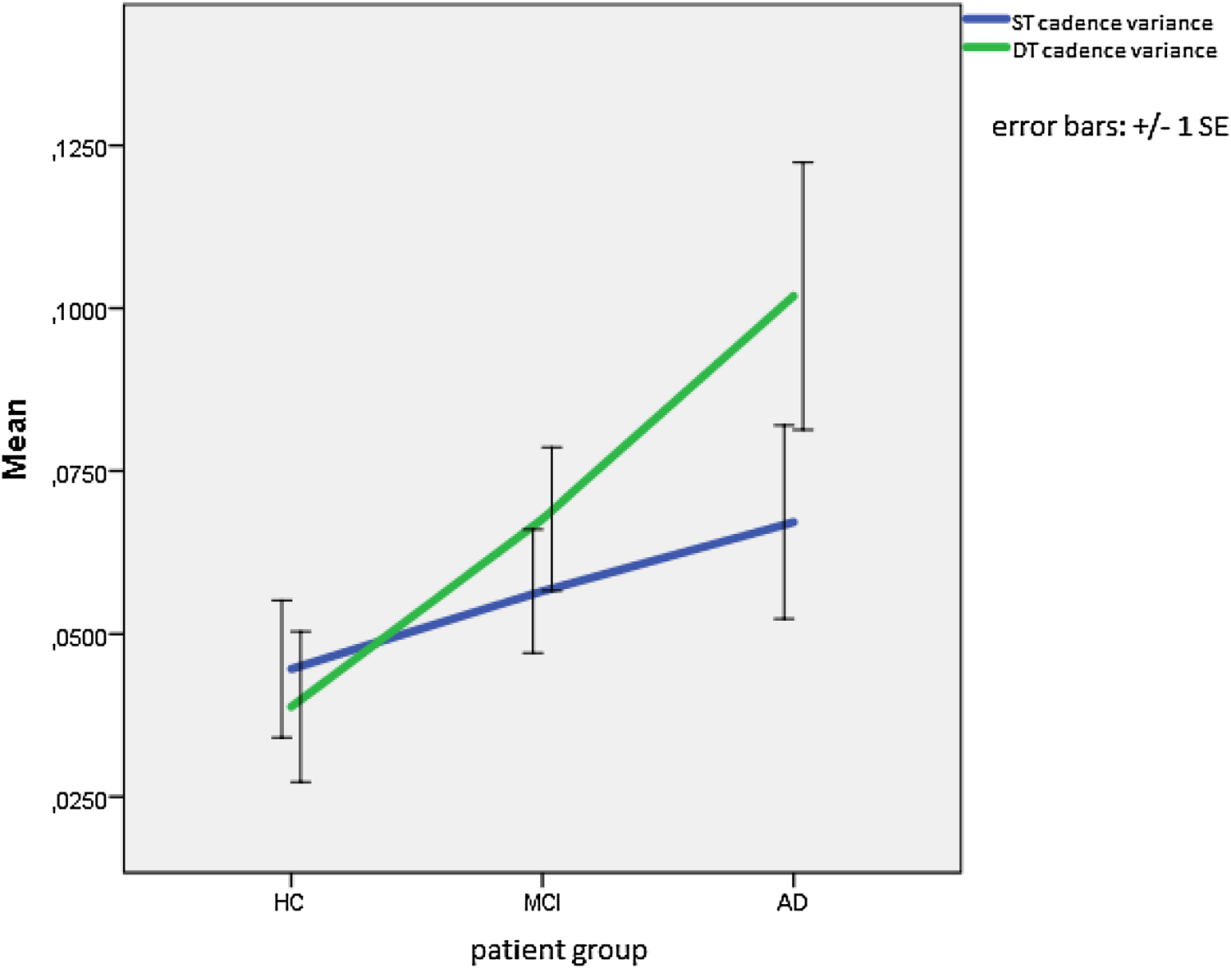
Step variance (seconds squared indicated on the Y axis) during the ST (*blue*) and the DT (*green*) *HC* healthy control subjects, *MCI* mild cognitive impairment subjects, *AD* Alzheimer’s disease subjects, *ST* single-task condition, *DT* dual-task condition

HC seem to have a smaller step variance and difference in step variance between ST and DT than MCI and AD patients (see Fig. 3). A mixed between-within ANOVA did, however, not find a significant main effect for step variance [Wilks’s Lambda = 0.97, F(1,65) = 1.73, *p* = .193, partial eta squared = 0.026]. There was a significant difference between groups [F(2,65) = 4.2, *p* = .019, partial eta squared = 0.115]. Post hoc tests revealed that the difference in step variance between the ST and DT differed between the HC [0.044 (± 0.05) vs. 0.039 (± 0.054)] and the AD group [0.067 (± 0.07) vs. 0.102 (± 0.099), *p* = .015] with the increase in step variance from the ST to the DT being greater for the AD patients. No or low correlations were found between DT step variance and neuropsychological measures of aspects of attention such as the MMSE subscale attention and calculation (*r* = −.211) and the TMT B (*r* = .348) or measures of motor performance such as the MMSE subscale complex commands (*r* = −.061).

## Discussion

The findings of this study add to the growing body of research on the interaction between cognitive function and motor performance and show that there are changes in gait parameters that may help distinguish healthy elderly from elderly with cognitive impairment. These changes were detectable with an actigraph which seems to be a useful tool combined with the dual-task paradigm for gait assessment in clinical practice. As mentioned previously, actigraphy has already been proven to be of interest for the evaluation of behavioral symptoms in dementia patients such as apathy [22] or agitation [23]. For example, recently, Valembois et al. investigated the value of wrist actigraphy as a measure of disorder in motor behavior in 183 elderly people with dementia finding that motor activity levels can distinguish dementia patients with apathy and dementia patients with aberrant motor behavior [24]. We were interested in the effect of performing a dual task on gait parameters given that dual tasking represents a cognitive challenge since it requests the allocation of attentional resources to concomitant tasks. Although we found differences between the single and dual tasks as well as between healthy elderly and AD patients, we only found significant differences between patient groups for walking speed and not for cadence and step variance. It seems that changes in gait induced by simultaneously performing a cognitive task between healthy elderly and individuals with MCI are so subtle that they are difficult to measure with an actigraph. The changes may become more salient and, thus easier to detect when patients progress to more severe stages of the disease. This is in line with previous findings. Schwenk et al. state in their review on gait parameters for frailty in elderly that gait speed showed the highest effect size to discriminate between different frailty status groups which suggests that this parameter is particularly informative and plays a significant role in gait analysis in elderly [37].

Although significant dual-task decrements have been demonstrated in AD [8, 10, 14], studies on the effects of dual tasking in MCI have not yield conclusive results. For instance, while Maquet et al. found reduced stride frequency and walking speed in MCI patients compared to healthy control subjects [15], Muir et al. did not find any gait differences between MCI patients and healthy control subjects [38]. These inconclusive results may be caused by several factors. First, the distance participants are asked to walk and the cognitive task they are asked to perform during dual tasking differ between studies. The Muir et al. study demonstrated that the dual task costs for two different cognitive tasks, i.e., naming animals and serial subtractions of seven, were comparable between AD and MCI patients. Nevertheless, it is possible that in the present study the cognitive task was too easy for participants with MCI and future research would benefit from using various cognitive tasks with variable difficulty levels. Second, the measure used to assess gait parameters as well as the algorithms used to analyze these parameters differ between studies. When it comes to actigraphy, the position of the placed accelerometer can have an important impact on reliability and quality of the measurement. As mentioned above, research has shown that a wrist-worn accelerometer can reliably distinguish between dementia patients with apathy and aberrant motor behavior and dementia patients who do not show these neuropsychiatric symptoms [24]. However, to measure gait parameters, more accurate measurements may be obtained when attaching the accelerometer to the participant’s waist, which was the case in the study of Maquet et al. Consequently, it is possible that the accelerometer on the participants’ wrist did not pick up subtle changes in gait parameters and is therefore not sensitive enough for the specific purpose of measuring gait during the performance of a dual task. An important limitation of our study is therefore the use of a wrist-worn accelerometer. A third explanation is that gait impairments in MCI patients are too small to detect with actigraphy and that the dual-task paradigm is not sensitive enough for early MCI screening [8, 16] but rather for more advanced stages [37]. As described above, changes may become more salient and, thus easier to detect when patients progress to more severe stages of the disease. It is interesting to mention in this regard that there was a higher variation in walking speed in the MCI group than in the HC and AD groups which can be explained by heterogeneity of the MCI group. It may therefore be valuable to make a better distinction within the MCI group, e.g., between non-amnestic and amnestic MCI patients, in future research. Related to this issue is the fact that many studies on dual tasking, including the present study, have a rather small sample size which may not explain finding significant differences in gait parameters between MCI patients and healthy elderly. Additionally, some of the participants may have suffered from vascular pathology for which it was difficult to control but which may explain the changes detected in the data.

Even though we did not find significant differences in dual tasking between HC and MCI patients, we believe that the findings of the present study warrant more research on the interaction between cognitive function and motor performance as an early indicator of cognitive decline. Future research would benefit from using a waist-worn rather than a wrist-worn accelerometer. Furthermore, we believe that future research would benefit from comparing a body-worn actigraph with other technologies used for gait analyses. As recently stated in the review by Schwenk et al., no standardized “sensitive technology exists for use within routine clinical care that would objectively quantify relevant gait parameters for indicating frailty status” [37]. As stated above, a more practical and low-cost solution for gait analysis would be very valuable for clinical practice. In addition, interesting topics for further research on the link between motor function and cognitive function in elderly with cognitive impairment include the relation between dual-task performance and an individual’s ability to carry out activities of daily living as a measure with higher ecologic validity. Moreover, it would be interesting to further explore the value of using wrist-worn accelerometers in a non-controlled environment to provide continuous information about subtle changes in walking parameters that could be useful to monitor progression of cognitive decline. In non-controlled environments, wrist-worn accelerometers may be preferred as they are more practical and less stigmatizing.

## Footnotes

1 Information about the MMSE complex commands subscale was only available for 46 participants.

2 When applying Bonferroni correction in order to account for multiple comparisons, the difference between groups for the registration subscale is no longer significant (for an alpha of 0.007).
